# A prospective cohort study of the long-term effects of CPAP on carotid artery intima-media thickness in Obstructive sleep apnea syndrome

**DOI:** 10.1186/1465-9921-13-22

**Published:** 2012-03-16

**Authors:** David S Hui, Qing Shang, Fanny W Ko, Susanna S Ng, Cheuk-Chun Szeto, Jenny Ngai, Alvin H Tung, Kin-Wang To, Tat-On Chan, Cheuk-Man Yu

**Affiliations:** 1 SH Ho Sleep Apnea Management Center, Department of Medicine & Therapeutics, The Chinese University of Hong Kong, Prince of Wales Hospital, 30-32 Ngan Shing St., Shatin, New Territories, Hong Kong

## Abstract

**Objective:**

To examine the long-term effect of CPAP on carotid artery intima-media thickness (IMT) in patients with Obstructive sleep apnea syndrome(OSAS).

**Methods:**

A prospective observational study over 12 months at a teaching hospital on 50 patients newly diagnosed with OSAS who received CPAP or conservative treatment (CT). Carotid IMT was assessed with B-mode Doppler ultrasound from both carotid arteries using images of the far wall of the distal 10 mm of the common carotid arteries at baseline, 6 months and 12 months.

**Measurements and results [mean (SE)]:**

Altogether 28 and 22 patients received CPAP and CT respectively without significant differences in age 48.8(1.8) vs 50.5(2.0)yrs, BMI 28.2(0.7) vs 28.0(1.2)kg/m2, ESS 13.1(0.7) vs 12.7(0.6), AHI 38(3) vs 39(3)/hr, arousal index 29(2) vs 29(2)/hr, minimum SaO_2 _75(2) vs 77(2)% and existing co-morbidities. CPAP usage was 4.6(0.3) and 4.7(0.4)hrs/night over 6 months and 1 year respectively. Carotid artery IMT at baseline, 6 months, and 12 months were 758(30), 721(20), and 705(20)micron for the CPAP group versus 760(30), 770(30), and 778(30)micron respectively for the CT group, p = 0.002.

Among those free of cardiovascular disease(n = 24), the carotid artery IMT at baseline, 6 months and 12 months were 722(40), 691(40), and 659(30)micron for the CPAP group (n = 12) with usage 4.5(0.7) and 4.7(0.7) hrs/night over 6 months and 12 months whereas the IMT data for the CT group(n = 12) were 660(20), 685(10), and 690(20)micron respectively, p = 0.006.

**Conclusions:**

Reduction of carotid artery IMT occurred mostly in the first 6 months and was sustained at 12 months in patients with reasonable CPAP compliance.

## Background

Obstructive sleep apnea syndrome (OSAS) is characterized by repetitive episodes of upper airway obstruction causing daytime sleepiness, impaired cognitive function and poor health status [1]. Untreated OSA is associated with increased risks of developing fatal and non-fatal cardiovascular events [2,3]. Three large prospective cohort studies have shown that untreated OSA is an independent risk factor for all-cause mortality after long-term follow-up [4-6]. Untreated OSA is also associated with dysglycemia, systemic inflammation, endothelial dysfunction, platelet activation, and other cardiovascular consequences such as cardiac arrhythmias especially atrial fibrillation (AF), coronary artery disease, asymptomatic early atherosclerosis, and silent brain infarction [7].

In recent years, carotid artery IMT, measured by B-mode ultrasound, has been shown to be a highly reproducible test and correlate well with traditional vascular risk factors. It may predict the likelihood of acute coronary events and stroke in asymptomatic healthy subjects [8,9]. Several studies have shown that the severity of OSA is independently related to the carotid artery IMT, with the severity of OSA-related hypoxemia more important than the frequency of obstructive events [10,11]. One randomized controlled trial (RCT) has shown that continuous positive airway pressure (CPAP) (n = 12) over 4 months could reduce carotid artery IMT in patients with severe OSAS free of existing cardiovascular diseases versus controls (n = 12) [12], but another recent RCT of 3 months treatment duration has failed to show any significant change in carotid artery IMT when comparing CPAP (n = 43) versus sham CPAP (n = 43) [13]. Hence it remains unknown whether CPAP can consistently reduce the carotid artery IMT in patients with OSAS or to a greater magnitude over a longer treatment period. This study examined the long-term effects of CPAP versus conservative treatment (CT) on carotid artery IMT over a period of 1 year.

## Methods

We conducted a prospective observational study of the treatment effects on carotid artery IMT in patients newly diagnosed with OSAS. OSAS, as defined by an overnight polysomnography (PSG) showing apnea-hypopnea index (AHI) ≥ 5/hour of sleep plus excessive daytime sleepiness or two of the following symptoms: choking or gasping during sleep, recurrent awakenings from sleep, unrefreshed sleep, daytime fatigue, and impaired concentration [14,15]. The patients were recruited from the Respiratory Clinic, Prince of Wales Hospital, Hong Kong. The inclusion criteria of the study included age 20 to 80 yrs, and AHI ≥5 hr on PSG with symptoms of OSA as described above. The exclusion criteria included patients having problems staying awake during driving, professional drivers, shift work, recent myocardial infarction, unstable angina, underlying malignancy, and treatment of hyperlipidemia with statins or other lipid-lowering agents. Our study was approved by the Ethics Committee of the Chinese University of Hong Kong (CRE-2005.135) and appropriate informed written consent was obtained from the subjects.

### Sleep assessment

Overnight diagnostic PSG (Healthdyne Alice 4, USA) was performed for every subject recording electroencephalogram(EEG), electro-oculogram, submental electromyogram (EMG), bilateral anterior tibial EMG, electrocardiogram, chest and abdominal wall movement by inductance plethysmography, airflow measured by a nasal pressure transducer [PTAF2, Pro-Tech, Woodinville, WA, USA] and supplemented by an oral thermister, and finger pulse oximetry as described in our previous studies [15,16]. Sleep stages were scored according to standard criteria by Rechtshaffen and Kales [17]. Apnea was defined as cessation of airflow for > 10 seconds and hypopnea as a reduction of airflow of ≥ 50% for > 10 seconds plus an oxygen desaturation of > 3% or an arousal. An arousal was scored if there was a 3 sec or longer abrupt shift in EEG frequency to alpha or theta or >16 Hz, following at least 10 sec of sleep, and if arising in REM there must be a rise in EMG tone [18].

Following confirmation of OSA, all patients were arranged to undergo an attended overnight autoCPAP titration on the second night of the sleep study. All patients were given a basic CPAP education program by our respiratory nurse supplemented by education brochure [15,16]. The nurse would fit a comfortable CPAP mask from a wide range of selection for every patient, who was then given a short trial of CPAP therapy with the Autoset (ResMed, Sydney, Australia) CPAP device for approximately 30 minutes for acclimatization in the afternoon. Following the overnight autoCPAP titration study, each patient was interviewed by the physician on duty and invited to participate in the serial carotid IMT study.

#### Group 1 (CT)

After confirmation of significant OSAS and completion of overnight attended autoCPAP titration, patients who were not keen to start CPAP yet were encouraged to a) avoid sleep deprivation by having sufficient hours of sleep every night; b) sleep in lateral positions; c) avoid sedatives and alcohol consumption 4 hours before sleep; and d) lose weight by exercise and diet where appropriate [19].

#### Group 2 (CPAP)

In addition to the usual advice as given to group 1, patients who had agreed to commence CPAP treatment after completing an overnight autoCPAP titration were subsequently prescribed CPAP device with a time counter recording machine run time. The CPAP pressure for each patient was set at the minimum pressure needed to abolish snoring, obstructive respiratory events, and airflow limitation for 95% of the night as determined by the overnight AutoSet CPAP titration study [15,16].

#### Carotid artery IMT

Was measured at baseline, 6 months, and 12 months for patients in both groups. The patients were followed up at the Respiratory clinic at 1, 3, 6 and 12, months whereas objective CPAP usage was measured from the time counter for group 2.

Carotid artery IMT was assessed by B-mode ultrasound scanning with an 11-MHz linear phase array transducer (Sonos 5500, Hewlett-Packard, Andover, Massachusetts, USA). Bilateral IMT measurements were obtained at the distal 10 mm of common carotid artery as described by our group previously [20,21]. The IMT was defined as the distance between the leading edge of the luminal echo to that of the media/adventitia echo and analyzed with a computerized edge-detection system (Q-Lab5.0, Xcelera, Phillips, USA). Three end-diastolic frames were selected, digitized, and analyzed for the mean IMT, and the average reading from these 3 frames was calculated for both right and left carotid arteries. The sole carotid scan operator (QS) was blinded to the clinical treatment status of the studied subjects and was not involved in the clinical assessment [20,21].

Blood pressure (BP) was measured in the right arm after at least 5 minutes of rest using a standard sphygmomanometer and the Korotkoff sound V was used as the indicator for the diastolic BP at baseline before PSG and at clinic visits at 6 months and 12 months.

### Statistical analysis

The sample size was estimated by the Power Analysis and Sample Size for Windows software (PASS 2000, NCSS, Kaysville, Utah). Based on the findings of Drager et al [12], group sample sizes of 28 would achieve 80% power to detect a difference of carotid IMT between the treatment and control groups (645 +/- 95 versus 740 +/−150 [micron]) at a significance level (alpha) of 0.05, using a two-sided paired Student's t test.

The primary end-point was the change in carotid artery IMT. For comparisons between the 2 groups at each time point, unpaired t-test was used for normally distributed variables and Mann-Whitney U test for non-normally distributed variables. To compare the measurements before and after CPAP treatment, paired t-test was used for normally distributed variables and Wilcoxon's signed rank test for non-normally distributed variables. Two-factor ANOVA (group versus time) with repeated measures on the factor time (baseline minus treatment) was used to test for the effect of CPAP versus CT. Data are expressed as mean ± SE unless stated otherwise. A p-value of < 0.05 is considered significant.

## Results

**Figure 1:**
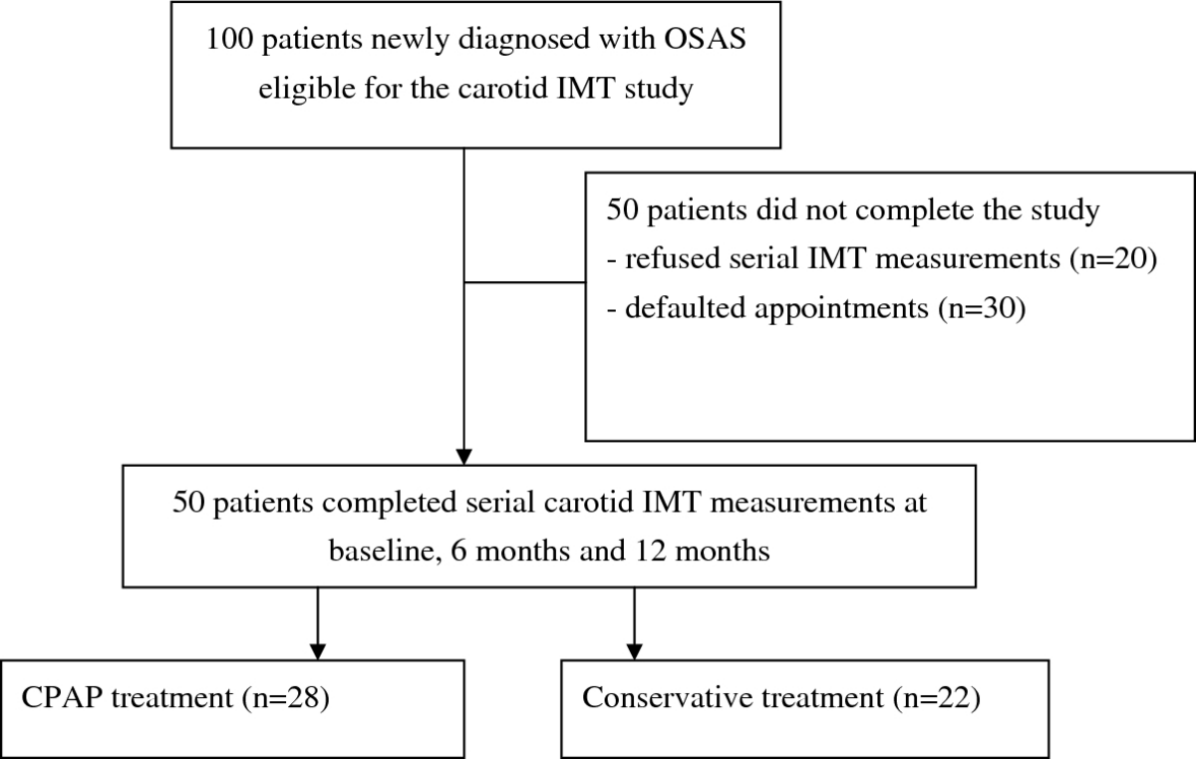
**Patient profile**. Among the 20 eligible patients who did not consent for the study, 14 had started CPAP treatment at home whereas 6 were subsequently referred for treatment with the mandibular advancement device. Among the 30 patients who had agreed to join the study initially, 22 were started on home CPAP whereas the other 8 opted for conservative treatment. However these 30 patients were unable to return for serial measurements of carotid IMT due to their busy work schedule.

We invited 100 patients with newly confirmed OSAS who had met the study criteria to participate in the serial carotid IMT study after completing PSG and an overnight autoCPAP titration. However, 50 eligible patients either refused to participate (n = 20) or could not take time off (n = 30) for completion of the serial carotid IMT study (Figure 1). There were no significant differences in demographics between patients who completed the carotid IMT study versus those who did not (Table 1). Among the remaining 50 patients who had completed the carotid IMT study, 22 received CT whereas 28 received CPAP treatment. The demographics and severity of OSA between the two groups were similar (Table 2). The subjects did not alter the dosage of their medications during the study.

**Table 1:** Comparisons of baseline parameters between patients who completed the 12-month carotid IMT study and those who did not

	Completed study (n = 50)	Did not complete study (n = 50)	P value
Male/Female	41/9	43/7	0.786

Age (yrs)	49.5 (1.4)	47.8 (1.7)	0.417

Body mass index (kg/m²)	28.1 (0.6)	28.2 (0.7)	0.954

Neck circumference (cm)	39.6 (0.5)	39.4 (0.5)	0.703

Systolic blood pressure (mmHg)	140.3 (3.1)	136.5 (3.1)	0.384

Diastolic blood pressure (mmHg)	80.6 (1.8)	79.8 (1.9)	0.749

Waist circumference (cm)	96.8 (1.6)	98.0 (1.7)	0.611

Hip circumference (cm)	101.6 (1.3)	102.6 (1.4)	0.602

Smoking status			
Non smoker	33	36	0.666
Smoker	17	14	

Alcoholic consumption			
Non drinker	23	21	0.840
Drinker	27	29	

ESS (0-24)	13.1 (0.7)	12.7 (0.6)	0.663

Congestive Heart Failure			
Yes	3	0	0.242
No	47	50	

Diabetes			
Yes	6	8	0.774
No	44	42	

Hypertension			

Yes	23	18	0.416
No	27	32	

Carotid plaque in baseline study			
Yes	12	10	0.810
No	38	40	

AHI (events per hr)	37.7 (3.0)	38.5 (3.4)	0.854

REM-AHI (events per hr)	35.9 (4.3)	33.1 (4.3)	0.652

Non-REM-AHI (events per hr)	37.8 (3.1)	38.6 (3.5)	0.878

Minimum SaO2 (%)	74.7 (2.2)	76.7 (2.2)	0.530

Mean SaO2 (%)	93.0 (0.6)	93.0 (0.6)	0.980

Arousal index (per hr of sleep)	29.3 (2.0)	28.8 (2.2)	0.857

Percentage of sleep time with SaO2<90%	13.7 (3.1)	7.5 (1.6)	0.081

Snoring/TST (%)	14.2 (3.9)	23.7 (4.0)	0.102

Sleep efficiency (%)	83.4 (1.5)	79.9 (1.8)	0.141

Mean Carotid IMT (micron)	758.6 (20)	742.6 (20)	0.573


Data expressed as mean (SE). TST = total sleep time

**Table 2:** Comparison of baseline parameters between patients on CPAP and Conservative treatment (CT)

	CPAP (n = 28)	CT (n = 22)	P value
Male/Female	25/3	16/6	0.157

Age (yrs)	48.8 (1.8)	50.5 (2.0)	0.526

Body mass index (kg/m²)	28.2 (0.7)	28.0 (1.2)	0.875

Neck circumference (cm)	40.5 (0.7)	38.5 (0.8)	0.060

Waist circumference (cm)	97.9 (1.9)	95.3 (2.8)	0.437

Hip circumference (cm)	102.2 (1.4)	100.8 (2.3)	0.589

Cholesterol (mmol/l)	5.8 (0.4)	5.2 (0.2)	0.180

HDL Cholesterol (mmol/l)	1.3 (0.1)	1.3 (0.1)	0.923

LDL Cholesterol (mmol/l)	3.3 (0.3)	3.0 (0.2)	0.449

Triglycerides (mmol/l)	3.3 (1.6)	2.2 (0.5)	0.477

Fasting plasma glucose (mmol/l)	5.2 (0.2)	5.5 (0.2)	0.223

Smoking status			
Non smoker	20	13	0.386
Smoker	8	9	

Alcoholic consumption			
Non drinker	14	9	0.577
Drinker	14	13	

ESS (0-24)	13.4 (0.9)	12.7 (1.0)	0.603

Congestive Heart Failure			
Yes	0	3	0.079
No	28	19	

Diabetes Mellitus			
Yes	3	3	1.000
No	25	19	

Hypertension			
Yes	14	9	0.577
No	14	13	

On anti-hypertensive drugs			
Yes	14	8	0.166
No	14	14	

On diabetic drugs			
Yes	2	1	1.000

No	26	21	

Carotid plaque in baseline study			
Yes	7	5	1.000
No	21	17	

Systolic blood pressure (mmHg)	140.8 (3.2)	139.7 (6.1)	0.873

Diastolic blood pressure (mmHg)	81.7 (2.1)	79.1 (3.4)	0.497

AHI (events per hr)	39.0 (3.6)	36.1 (5.1)	0.633

REM-AHI (events per hr)	39.1 (6.5)	32.5 (5.7)	0.459

Non-REM-AHI (events per hr)	39.0 (3.7)	36.2 (5.3)	0.662

Minimum SaO2 (%)	74.7 (2.1)	74.8 (4.2)	0.983

Mean SaO2 (%)	93.7 (0.4)	92.1 (1.2)	0.209

Arousal index (per hr of sleep)	31.1 (2.4)	27.1 (3.4)	0.331

Percentage of sleep time with SaO2<90%	11.1 (2.7)	17.0 (6.3)	0.399

Snoring/TST (%)	11.8 (4.6)	18.5 (7.7)	0.435

Sleep efficiency (%)	82.5 (2.0)	84.5 (2.3)	0.513

Mean carotid IMT (micron)	757.5 (30)	760.0 (30)	0.952


Data expressed as mean (SE)

### Comparisons of changes of parameters between CPAP group and CT group

**Figure 2:**
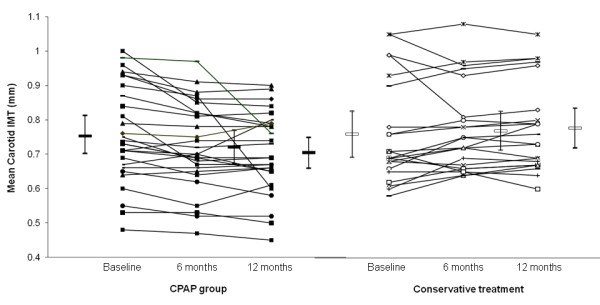
**Shows the data plots of OSA patients on CPAP (n = 28) and those on conservative treatment (n = 22)**. The serial mean carotid artery IMT at baseline, 6 months and 12 months were 757.5(30), 720.9(20) and 704.5(20)micron for the CPAP group versus 760.0(30), 769.8(30), and 777.7(30)micron respectively for those on conservative treatment, p = 0.002 (ANOVA for repeated measures).

The objective CPAP usage were 4.6(0.3) and 4.7(0.4) hrs/night for the CPAP group over 6 months and 1 year respectively. The serial mean carotid artery IMT at baseline, 6 months and 12 months were 757.5(30), 720.9(20) and 704.5(20)micron for the CPAP group (Figure 2) whereas the serial IMT data for the CT group were 760.0(30), 769.8(30), and 777.7(30)micron respectively, p = 0.002 (ANOVA for repeated measures) (Table 3 and Figure 2).

**Table 3:** Repeated measures comparisons of serial parameters between CPAP and CT at baseline, 6 months and 12 months

		CPAP (n = 28)			CT (n = 22)		P-value
	
	Baseline	6 m	12 m	Baseline	6 m	12 m	
**Neck circumference (cm)**	40.5 (0.7)	40.2 (0.6)	40.2 (0.7)	38.5 (0.8)	38.2 (0.8)	38.5 (0.8)	0.819

**Waist circumference (cm)**	97.9 (1.9)	98.0 (1.6)	97.9 (1.7)	95.3 (2.8)	93.2 (2.9)	94.7 (2.9)	0.260

**Hip circumference (cm)**	102.2 (1.4)	104.0 (1.5)	105.2 (1.3)	100.8 (2.3)	102.5 (2.2)	102.7 (2.1)	0.653

**Body mass index (kg/m²)**	28.2 (0.7)	28.6 (0.7)	28.3 (0.8)	28.0 (1.2)	27.7 (1.1)	27.9 (1.2)	0.042

**Systolic BP (mmHg)**	140.8 (3.2)	129.0 (3.6)	130.7 (3.6)	138.4 (5.4)	135.9 (5.4)	136.1 (5.2)	0.593

**Diastolic BP (mmHg)**	81.7 (2.1)	82.3 (2.1)	83.3 (2.1)	78.6 (3.0)	84.9 (2.9)	83.8 (3.7)	0.412

**ESS (0-24)**	13.4 (0.9)	8.4 (0.8)	8.2 (1.0)	12.7 (1.0)	10.2 (1.2)	10.3 (1.5)	0.110

**CPAP usage (hrs/night)**	N/A	4.6 (0.3)	4.7 (0.4)	N/A	N/A	N/A	N/A

**Mean carotid IMT (micron)**	757.5 (30)	720.9 (20)	704.5 (20)	760.0 (30)	769.8 (30)	777.7 (30)	0.002


Values are expressed as mean (SE)

The p-values of BP trends from baseline to 12 months within the CPAP group are as follows: systolic BP, p = 0.013; diastolic BP, p = 0.573

The changes in mean carotid artery IMT between baseline and 6 months were −36.6 (10) versus 9.8(10)micron for the CPAP and CT group respectively, 95%CI (−77, −15.8 micron), p = 0.004. The changes in mean carotid artery IMT between baseline and 12 months were −53(20) versus 17.7(10) micron for the CPAP and the CT group respectively, 95%CI (−114.8, −26.7 micron), p = 0.002. The changes in mean carotid artery IMT between 6 months and 12 months were −16.4(10) versus 8(10) micron for the CPAP and the CT group respectively, 95%CI (−56, 72.2 micron), p = 0.127.

There was no correlation between objective CPAP usage and changes in carotid IMT at 6 months, r = −0.185 (p = 0.375) and at 12 months, r = −0.018 (p = 0.930).

### Comparisons of changes of parameters between CPAP group and CT group among those free of existing cardiovascular disease (excluding smoking and alcohol intake)

**Figure 3:**
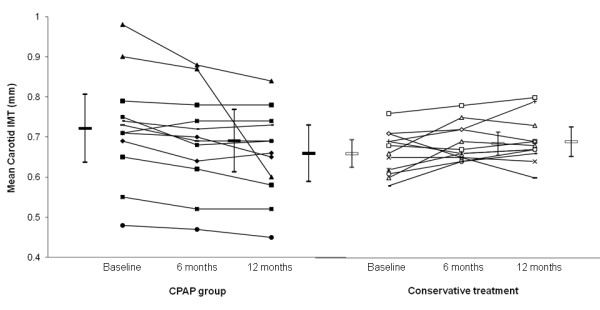
**Shows the data plots of OSA patients free of existing cardiovascular diseases (excluding smoking and alcohol consumption)**. The mean carotid artery IMT at baseline, 6 months and 12 months were 721.7(40), 690.8(40) and 659.2(30) micron for the CPAP group (n = 12) versus 660.0(20), 684.6(10) and 690.0(20)micron respectively for those on conservative treatment (n = 12), p = 0.006 (ANOVA for repeated measures).

Among those free of existing cardiovascular disease (n = 24), there were no significant differences in baseline demographics and severity of OSAS between the two groups including the absence of carotid plaques (Table 4). The mean carotid artery IMT at baseline, 6 months and 12 months were 721.7(40), 690.8(40), and 659.2(30) micron for the CPAP group (n = 12) with objective CPAP usage 4.5(0.7) and 4.7(0.7) hrs/night over 6 months and 12 months (Figure 3) whereas the corresponding carotid IMT data for the CT group (n = 12) were 660.0(20), 684.6(10) and 690.0(20) micron respectively, p = 0.006 (ANOVA for repeated measures) (Table 5 and Figure 3).

**Table 4:** Comparisons of baseline parameters of patients free of any cardiovascular co-morbidity (excluding smoking and alcohol consumption) who completed serial carotid IMT measurements between CPAP and CT group

	CPAP (n = 12)	CT (n = 12)	P value
Male/Female	11/1	9/3	0.590

Age (yrs)	44.3 (2.1)	48.5 (1.7)	0.135

Body mass index (kg/m²)	27.4 (1.1)	25.9 (1.1)	0.357

Neck circumference (cm)	40.0 (1.1)	37.6 (1.0)	0.127

Waist circumference (cm)	96.3 (3.2)	91.9 (2.9)	0.320

Hip circumference (cm)	101.7 (2.2)	97.5 (2.1)	0.179

Systolic blood pressure (mmHg)	139.9 (3.6)	129.7 (6.5)	0.164

Diastolic blood pressure (mmHg)	81.7 (2.7)	75.7 (3.2)	0.160

Cholesterol (mmol/l)	5.8 (0.5)	5.4 (0.4)	0.535

HDL Cholesterol (mmol/l)	1.5 (0.3)	1.2 (0.1)	0.331

LDL Cholesterol (mmol/l)	3.7 (0.4)	3.4 (0.3)	0.548

Triglycerides (mmol/l)	1.3 (0.3)	1.8 (0.3)	0.338

Fasting plasma glucose (mmol/l)	5.2 (0.3)	5.1 (0.1)	0.676

ESS (0-24)	12.7 (1.0)	11.5 (1.3)	0.477

Smoking status			
Non smoker	10	8	0.640
Smoker	2	4	

Alcoholic consumption			
Non drinker	7	5	0.684
Drinker	5	7	

AHI (events per hr)	36.3 (6.5)	35.5 (6.6)	0.929

REM-AHI (events per hr)	34.4 (6.5)	30.5 (7.6)	0.712

Non-REM-AHI (events per hr)	36.4 (6.8)	35.7 (6.7)	0.942

Minimum SaO2 (%)	77.0 (3.4)	80.0 (2.9)	0.509

Mean SaO2 (%)	94.2 (0.7)	93.9 (0.9)	0.827

Arousal index (per hr of sleep)	29.1 (4.8)	26.8 (5.1)	0.746

Percentage of sleep time with SaO2<90% (%)	10.3 (4.7)	7.6 (3.2)	0.645

Snoring/TST (%)	16.2 (7.7)	7.0 (3.2)	0.510

Sleep efficiency (%)	81.9 (3.0)	81.6 (3.7)	0.943

Mean carotid IMT (micron)	721.7 (40)	660.0 (20)	0.153


**Table 5:** Repeated Measures - Comparisons of serial parameters between CPAP and CT at baseline, 6 months and 12 months

		CPAP (n = 12)			CT (n = 12)		P-value
	
	Baseline	6 m	12 m	Baseline	6 m	12 m	
**Neck circumference (cm)**	40.0 (1.1)	39.2 (0.9)	39.4 (1.0)	37.6 (1.0)	37.4 (1.0)	37.5 (1.1)	0.516

**Waist circumference (cm)**	96.3 (3.2)	94.3 (3.3)	94.6 (3.5)	91.9 (2.9)	88.7 (3.6)	91.1 (3.5)	0.527

**Hip circumference (cm)**	101.7 (2.2)	102.6 (2.5)	104.2 (2.3)	97.5 (2.1)	98.8 (2.3)	100.4 (2.6)	0.967

**Body mass index (kg/m²)**	27.4 (1.1)	27.6 (1.1)	27.4 (1.1)	25.9 (1.1)	25.9 (1.1)	26.0 (1.2)	0.723

**Systolic BP (mmHg)**	139.9 (3.6)	118.0 (5.0)	121.7 (4.7)	128.9 (5.7)	124.8 (4.7)	126.8 (6.8)	0.108

**Diastolic BP (mmHg)**	81.7 (2.7)	76.9 (3.8)	80.0 (3.4)	75.2 (2.9)	82.4 (3.9)	81.5 (5.3)	0.219

**ESS (0-24)**	12.7 (1.0)	7.3 (1.3)	6.4 (1.2)	11.5 (1.3)	8.7 (1.4)	8.6 (1.6)	0.145

**CPAP usage (hrs/night)**	N/A	4.5 (0.7)	4.7 (0.7)	N/A	N/A	N/A	N/A

**Mean carotid IMT (micron)**	721.7 (40)	690.8 (40)	659.2 (30)	660.0 (20)	684.6 (10)	690.0 (20)	0.006


Values are expressed as mean (SE)

The p-values of BP trends from baseline to 12 months within the CPAP group are as follows: systolic BP, p < 0.001; diastolic BP, p = 0.517

The changes in mean carotid artery IMT between baseline and 6 months were −30.8 (10) versus 24.6(10) micron for the CPAP and CT group respectively, 95%CI (−89.4, −21.4 micron), p = 0.003. The changes in mean carotid artery IMT between baseline and 12 months were −62.5(20) versus 30(20) micron for the CPAP and CT group respectively, 95%CI (−155.4, −29.6 micron), p = 0.006. The changes in mean carotid artery IMT between 6 months and 12 months were −31.7(20) versus 5.4(10) micron for the CPAP and the CT group respectively, 95%CI (−87.9, 13.7 micron), p = 0.144.

## Discussion

In a group of 50 symptomatic patients newly diagnosed with severe OSAS, this prospective observational study has shown that CPAP treatment (n = 28) resulted in significant reduction in carotid artery IMT compared to those who had opted for conservative treatment (CT, n = 22) over a study period of 12 months. Most of the reduction in carotid artery IMT when comparing CPAP against CT group appeared to have occurred within the first 6 months of treatment whereas there was no significant change from 6 to 12 months while the patients had maintained reasonable CPAP usage objectively throughout the study. Similar observations were noted in patients with and without existing cardiovascular diseases.

Data from the Sleep Heart Health Study (SHHS) have shown that modest to severe levels of OSA are associated with an approximately threefold increased risk of ischemic stroke in community-dwelling men [22]. The Wisconsin Sleep Cohort Study has provided prospective evidence that OSA is related to significantly increased odds of suffering a stroke over the next 4 years after adjustment for age and gender [23]. In an observational sleep clinic study, Yaggi et al [24] have shown that OSA significantly increases the risk of stroke or death from any cause and the increase is independent of other known risk factors. Patients with stroke and OSA have an increased risk of early death over 10 years [25], whereas sleep apnea is significantly associated with increased risk of stroke among patients with coronary artery disease over a follow-up period of 10 years [26].

There are several proposed mechanisms linking OSA and stroke. Snoring-induced vibrational injury may lead to carotid atherosclerosis [27]. There is a strong association between OSA and AF [28]. Platelet activation [16] and silent brain infarction were also more common in patients with moderate to severe OSA than in controls [29]. OSA may accelerate atherosclerosis through the effect of hypertension and other mechanisms such as insulin resistance, diabetes, and dyslipidemia. In addition, OSA can induce direct proatherogenic effects through the mechanisms of systemic inflammation, oxidative stress, vascular smooth cell activation, increased adhesion molecule expression, monocyte/lymphocyte activation, increased lipid loading in macrophages, lipid peroxidation, and endothelial dysfunction [30].

In recent years, carotid artery IMT has been well accepted as a non-invasive tool which may predict the likelihood of acute coronary events and stroke in asymptomatic healthy subjects [8,9]. Carotid artery IMT has been applied by several research groups to study different OSA populations. Although cross-sectional analysis of the SHHS has found no evidence that mild to moderate SDB is associated with subclinical atherosclerosis [31], data from other groups have suggested that OSA may lead to early atherosclerosis, as reflected by increase in carotid artery IMT and occurrence of plaques, in the absence of any significant comorbidity [32-34]. In one series of OSA patients, severity of oxygen desaturation and BP status were the best predictors for carotid wall hypertrophy whereas plaque occurrence without known cardiovascular disease was also related to the amount of oxygen desaturation regardless of their BP status [33]. OSA-related hypoxia and systemic inflammation might be associated with progression of atherosclerosis and increased risk of cardiovascular morbidity [34]. Another study has demonstrated a relationship between lipid peroxidation, carotid artery IMT, and intermittent hypoxia in non-obese OSA patients [35] whereas in patients with minimally symptomatic OSA, diverse properties of endothelial function are impaired and arterial stiffness is increased [36]. To date, only one RCT with a small sample size has shown that CPAP therapy (n = 12) over 4 months could reduce carotid IMT in patients with severe OSAS free of existing cardiovascular diseases versus controls (n = 12) (mean changes of −62 vs 8 micron for the two groups respectively, p = 0.02) [12].

In this study, there were significant differences when comparing the changes in carotid IMT at 6 months [-36.6 (10) versus 9.8(10)micron] and at 12 months [-53(20) versus 17.7(10) micron] respectively from baseline between CPAP and CT groups. The magnitude of reduction in carotid IMT with CPAP was similar to those patients with OSAS with and without existing cardiovascular disease who received CPAP treatment. A clinical trial comparing rosuvastatin vs placebo among 984 low risk subjects showed no significant difference in the rate of mean maximum carotid IMT progression after 6 months (2.3 vs 10.6 micron/year, p = 0.34). However, carotid IMT progression rates were significantly different when comparing rosuvastatin vs placebo at 12 months, (3.2 vs 13.3 micron/year, p = 0.049) whereas the divergence grew with further follow-up (−0.9 vs 13.1 micron/year at 18 months and -1.4 vs 13.1 micron/year after 24 months of treatment, p < 0.001 for both time points) [37].

Although we did not find any significant correlation between objective CPAP usage and carotid IMT in this study, variability in the individual response may be related to the severity of OSA (AHI, hypoxemia) and CPAP compliance. Although the changes in carotid IMT with CPAP (n = 43) versus sham CPAP (n = 43) were not significant in the whole study population by Sharma et al [13], a subgroup analysis among those (n = 51) with CPAP usage at least 5 hrs/night showed significant reduction in carotid IMT (34 vs 14 micron, p < 0.05) when comparing CPAP vs sham CPAP treatment over 3 months.

This study is limited by the fact that it was not a RCT as it would not be ethical to withhold CPAP treatment for symptomatic patients with severe OSAS for a 1-year study in our locality. Only 50% of eligible OSA patients had participated in this study although the demographics and severity of OSA between those who participated in this study were similar to those who did not. Likewise patients who received CPAP and those who opted for CT were similar in terms of demographics and baseline severity of OSA. Lastly only baseline data of glucose, lipids and carotid plaques were available and we did not have serial data to assess the treatment effects.

## Conclusion

In summary, this prospective observational study has shown that CPAP treatment resulted in significant reduction in carotid artery IMT whereas no significant change was noted among those who opted for conservative treatment over a study period of 1 year. Reduction in carotid artery IMT within the CPAP group occurred mostly within the first 6 months of treatment in patients with and without existing cardiovascular diseases and the reduction well maintained at 12 months in patients with reasonable CPAP compliance. Patients newly diagnosed with OSAS should be encouraged to comply with CPAP not just to relieve daytime sleepiness but there may be cardio-protective effects. Further studies with the RCT design over short to medium term are warranted to assess the effect of CPAP on carotid IMT.

## Abbreviations

AF: Atrial fibrillation; AHI: Apnea-hypopnea index; CPAP: Continuous positive airway pressure; CT: Conservative treatment; ESS: Epworth sleepiness score; IMT: Intima-media thickness; OSAS: Obstructive sleep apnea syndrome; PSG: Polysomnography; TST: Total sleep time.

## Competing interests

The authors declare that they have no competing interests.

## Authors' contributions

DSH was the guarantor responsible for the study conception and design, data interpretation and writing of the manuscript. QS was responsible for the carotid IMT measurement. CCS and CMY were responsible for interpretation of the carotid IMT data and critical revision of the manuscript. FWK, JN, SSN, AHT, TOC and KWT were responsible for interpretation of sleep study and clinical assessment of patients. All authors have read and approved the submitted manuscript.
